# Measurements of signal intensity of globus pallidus and dentate nucleus suggest different deposition characteristics of macrocyclic GBCAs in children

**DOI:** 10.1371/journal.pone.0208589

**Published:** 2018-12-26

**Authors:** Cyprian Olchowy, Ewa J. Maciąg, Angel Sanchez-Montanez, Anna Olchowy, Ignacio Delgado, Elida Vazquez

**Affiliations:** 1 Department of Oral Surgery, Wroclaw Medical University, Wrocław, Poland; 2 Pediatric Radiology Department, Vall d'Hebron University Hospital, Barcelona, Spain; 3 Department of Experimental Dentistry, Wroclaw Medical University, Wrocław, Poland; McLean Hospital, UNITED STATES

## Abstract

**Introduction:**

The safety of using GBCAs to enhance the visibility of body structures is currently discussed due to possible gadolinium retention in brain structures. The aim of the study was to evaluate the effect of multiple exposures to macrocyclic GBCAs in children.

**Materials and methods:**

This retrospective, single-center study included data from 43 patients who had received ≥4 injections of macrocyclic GBCAs during MRI examinations over performed over 8 to 84 months. Signal intensity was measured on unenhanced T1-weighted MRI, and globus pallidus to thalamus (GP/Th) and dentate nucleus to pons (DN/P) ratios were calculated. The differences in ratios were tested with the Student’s t-test or the Wilcoxon rank sum test. For categorical data, Pearson's chi-squared test was used. Relationships were analyzed with the Spearman's rank correlation coefficient.

**Results:**

Patients with the mean age of 7.5 years (SD = 4.2) received 8.19 (SD = 3.63) injections of GBCAs on average. Differences in GP/Th and DN/P ratios between the first and the last measurement were insignificant. Children before the end of myelination process (≤2 years of age) had the first GP/Th ratio values significantly lower than those >2 years of age (p = 0.0284), which than increased at the final scan and reached the level similar to values obtained in the group of >2 years of age.

**Conclusions:**

Maturation of the brain may affect both signal intensity of brain structures and susceptibility to GBCAs; thus, assessment of signal intensity of the brain structures should be conducted taking into account the age of a child.

## Introduction

Magnetic resonance imaging (MRI) is one of the most valuable noninvasive diagnostic modalities in clinical practice. Although this technique is considered safe and accurate, the safety of using gadolinium based contrast agents (GBCAs) to enhance the visibility of body structures is currently widely discussed. Gadolinium belongs to the group of rare-earth metals and has got paramagnetic properties. The high magnetic moment and relatively long magnetic relaxation time is the rationale for using this element in diagnostic imaging. The high toxicity of free gadolinium was eliminated by closing free Gd3+ions in organic chelates [1].

Although GBCAs have been used in diagnostic imaging since 1988, only 4 years passed since Kanda et al. reported the association between a considerable increase in signal intensity within certain areas of the brain on MRI scans following multiple injections of gadolinium-based contrast agents [2]. Much new information has been gathered since that time. Several studies confirmed the increase in signal intensity of certain brain structures; however, the majority of them included adult patients. Only few studies and case reports addressed possible effect of gadolinium agent administration on brain structures in children [3–8]. Generally, the association between increased signal intensity on T1-weighted images was linked to the stability of the administered GBCA. Repeated administrations of linear GBCAs result in an increase in the signal intensity. Although, this effect is stronger after injections of linear ionic GBCAs than linear nonionic GBCAs (e.g. gadobenate dimeglumine) [9–13], signal intensity increases in case of both contrast agents [10, 14]. This phenomenon is rarely observed after repeated administration of macrocyclic GBCAs, except few recently published studies on pediatric population [4, 8, 9, 11, 12, 15]. Some of the studies found associations between the signal intensity of the brain structures with a cumulative dose of the contrast agent and the number of contrast injections. Additionally, the evidence that the increase in signal intensity reflects gadolinium depositions was provided by animal studies and autopsy examinations [16–18]. The summary of recent studies demonstrated that the presence of signal intensity is mainly associated with the type of a contrast agent [19]. Therefore, radiologists opt for the use of macrocyclic GBCAs to prevent accumulation of gadolinium compounds in the brain, because those agents are considered to be more stable. Taking into account pediatric population, the level of available knowledge is still insufficient. Although, we know that macrocyclic GBCAs are more stable what may translate into their safety, the development of gadolinium depositions of those macrocyclic GBCAs are possible. Additionally, the association between brain maturation and susceptibility to GBCA remains unknown. The need for pediatric data is especially urgent because there is a linear GBCA approved for the use in newborns in the US. For this reason, we aimed to evaluate the effect of serial injections of the macrocyclic GBCAs on the signal intensity (SI) change in the dentate nucleus and globus pallidus as measured by dentate nucleus to pons (DN/P) and globus pallidus to thalamus (GP/Th) ratio on unenhanced T1-weighted MR images in pediatric population.

## Methods and materials

### Patients

This was the retrospective, single-center study of data including 43 patients treated for cancer who had undergone repeated injections of macrocyclic GBCAs (gadoterate meglumine, gadobutrol). The study was approved by the Ethics Committee of the Vall d’hebron University Hospital. The requirement to obtain informed consent was waived. Data from patients were retrieved from our institution’s electronic database and checked whether they meet the inclusion criteria and exclusion criteria.

For the study, we selected patients aged below 18 years who had at least 4 administrations of contrast agent-enhanced brain MR examinations with a macrocyclic GBCA (gadoterate meglumine, gadobutrol) and normal MR brain findings on the most recent MR scan. Regarding contrast agents, patients who received both or single type of macrocyclic GBCA were included. We did not include patients who received any linear GBCA in the past, had a history of brain radiation as well as presented with impaired renal or hepatic function.

### Data collection

The MRI examinations were performed in 1 center using the same parameters of sequences (T1 spin echo sequence TR 400, TE 9.7) with 1.5-T scanner (Magnetom Avanto; Siemens Medical Systems, Erlangen, Germany). Signal intensity was measured in a circular region of interest (average diameter of 5 mm) within the globus pallidus, thalamus, dentate nucleus, and pons. Measurements of the right globus pallidus, right dentate nucleus, right thalamus, and the pons were taken on unenhanced axial T1-weighted MR images. Three measurements of each structure were taken and presented as the mean value. Next, GP/Th and DN/P ratios were calculated. All measurements were performed by a radiologist with 8 years of experience in pediatric neuroimaging and, in case of doubts, consulted with a second radiologist who performed additional measurements.

For the purpose of the study, patients were divided into two groups: children who had the first contrast enhanced MRI at the age of below 2 years (before myelination is finished) and those who had the first contrast enhanced MRI at the age of above 2 years.

### Statistical analysis

Summary values were given as means (standard deviation of the mean). The normality of distribution was tested using the Shapiro-Wilk normality test. As the analyzed variables had a normal distribution, the comparison between two groups of variables was conducted with the Student’s t-test, otherwise with the Wilcoxon rank sum test. For categorical data, Pearson's chi-squared test was used. Relationships were analyzed with the Spearman's rank correlation coefficient. Data were considered to be statistically significant at a value of p<0.05. Statistical analysis was carried out with the R Project for Statistical Computing v. 3.2.2.

## Results

The data from 43 pediatric patients were analyzed. The mean age of the study group was 7.5 (SD = 4.17). They had a history of 8.3 (SD = 3.73) contrast examinations on average ranging from 4 to 17 performed over 8 to 84 months. In all patients, serial MRIs were performed to monitor cancer disease. Patients were mainly diagnosed with tumors of WHO grade 1 such as pilocytic astrocytoma (16 cases), hairy cell leucaemia (9 cases), ganglioglioma (3 cases), dysembryoplastic neuroepithelial tumor (3 cases) and 1 case of gangliocytoma, and ganglioma, glioma. WHO grade 2 tumors, such as pleomorphic xanthoastrocytoma and fibrillary astrocytoma were diagnosed in 3 cases in total. Additionally, 6 patients had tuberous sclerosis. None of the patients presented with involvement of posterior cranial fossa and basal ganglia by primary cancer disease. None of the patient had been or was treated with surgery. Patient clinical characteristics along with the average values of analyzed parameters are presented in Table 1.

**Table 1:** Characteristics of the study group by age.

Variable	Total	First scan ≤2 years	First scan >2 years	p value
Number of patients	43	6	37	-
Age at first MRI; yrs	7.5 (4.20)	1.3 (0.82)	8.5 (3.61)	-
No. of enhanced MRI	8.19 (3.63)	7.5 (1.52)	8.3 (3.86)	0.38
Interval between first and last MRI (mth)	55 (22.50)	36 (9.87)	58 (22.65)	**0.02**
Interval between GBCA scans (mth)	7.46 (4.02)	5.0 (1.46)	7.8 (4.17)	0.14
First GP/Th ratio	1.04 (0.10)	0.96 (0.09)	1.05 (0.10)	**0.03**
Last GP/Th ratio	1.06 (0.08)	1.06 (0.09)	1.06 (0.08)	0.99
Δ GP/Th	0.03 (0.11)	0.11 (0.11)	0.01 (0.11)	0.06
First DN/P ratio	0.92(0.09)	0.90 (0.11)	0.99 (0.09)	0.72
Last DN/P ratio	0.93 (0.11)	0.88 (0.09)	0.94 (0.12)	0.22
Δ DN/P	0.01 (0.12)	-0.02 (0.14)	0.01 (0.12)	0.60
No. of radiotherapy treatments	0	0	0	-
No. of chemotherapy treatments	14	3	11	0.33
Parenteral nutrition	0	0	0	-

Data are presented as means (standard deviation)

**Fig 1:**
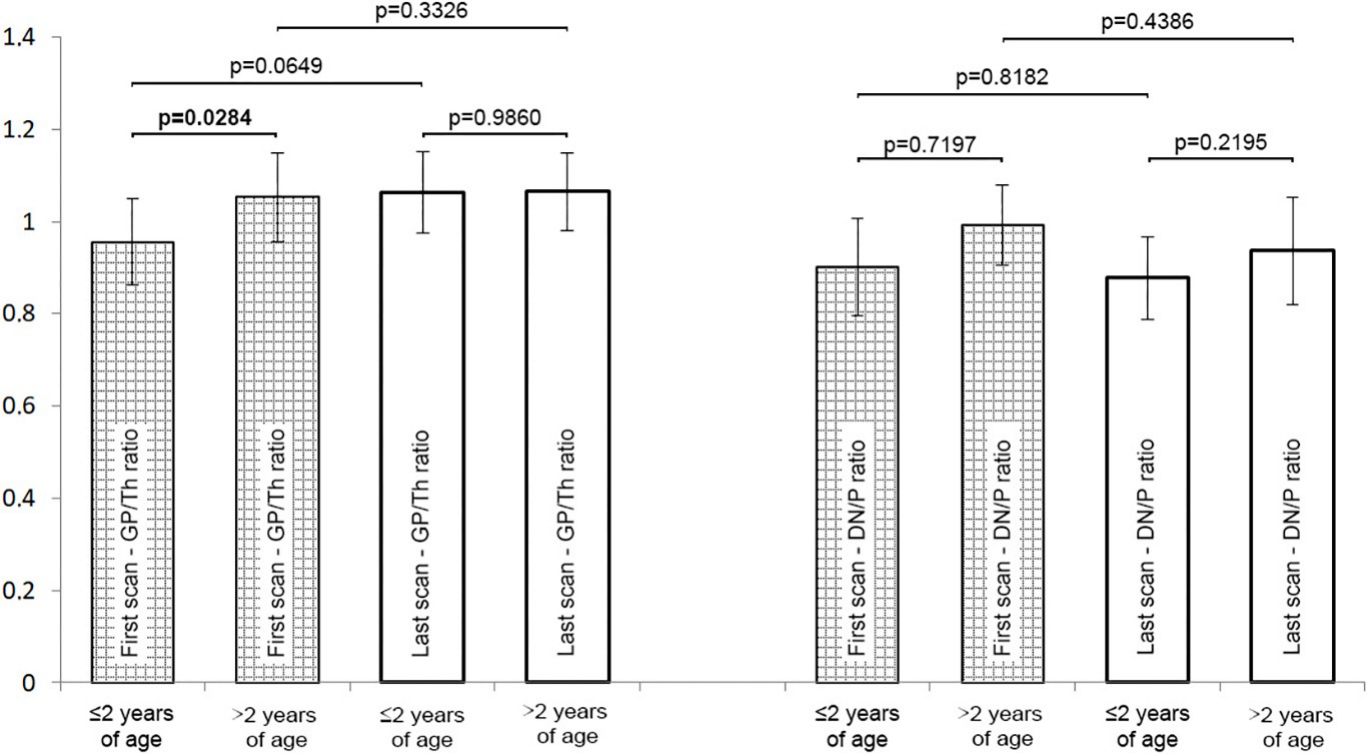
Comparison of GP/Th ratio and the DN/P ratio in the study group by age.

No significant differences were found between the first and the last measurement in terms of both the GP/Th ratio (p = 0.10) and the DN/P ratio (p = 0.70). We divided the study by age. The cut-off level was set at the end of the myelination process at 2 years of age. In terms of clinical characteristics, there were no significant differences between children aged ≤2 years and above 2 years except for the first GP/Th ratios which was significantly lower in younger children than older ones as well as treatment duration which was shorter in younger children than older ones. In terms of signal intensity, the only statistical difference in GP/Th ratio was found between the first measurement in younger and older children (Fig 1).

**Fig 2:**
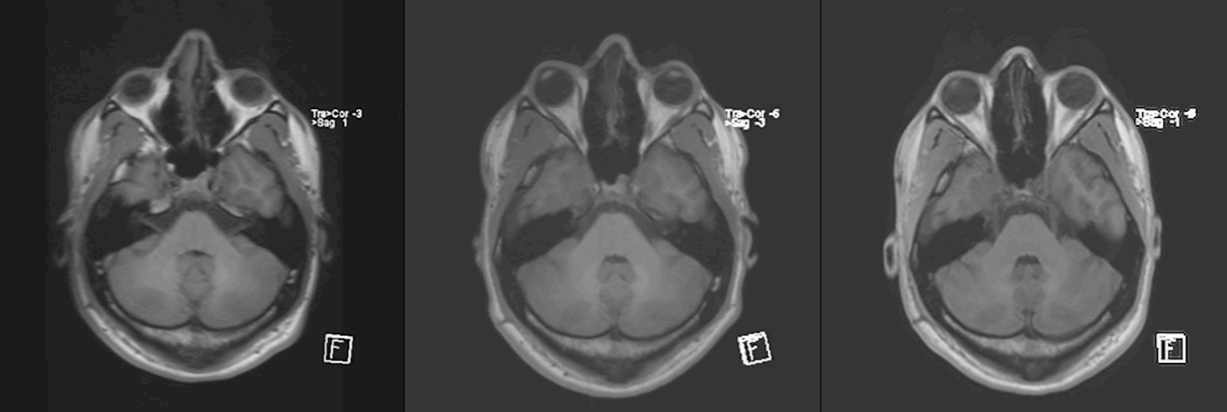
A male patient with pilocytic astrocytoma aged 9 at the first examination after 7 injections of macrocyclic GBCAs; the following scans show the baseline conditions, a scan after the 4^th^ injection of the contrast agent and final scan.

Some scans presented an impression of a visual increase in the signal intensity which could potentially have an effect on the dentate nucleus to pons ratio on MRI scans; an example of such an increase is shown in Fig 2; however, statistical analysis did not confirm significant differences between the first and the last measurements.

**Fig 3:**
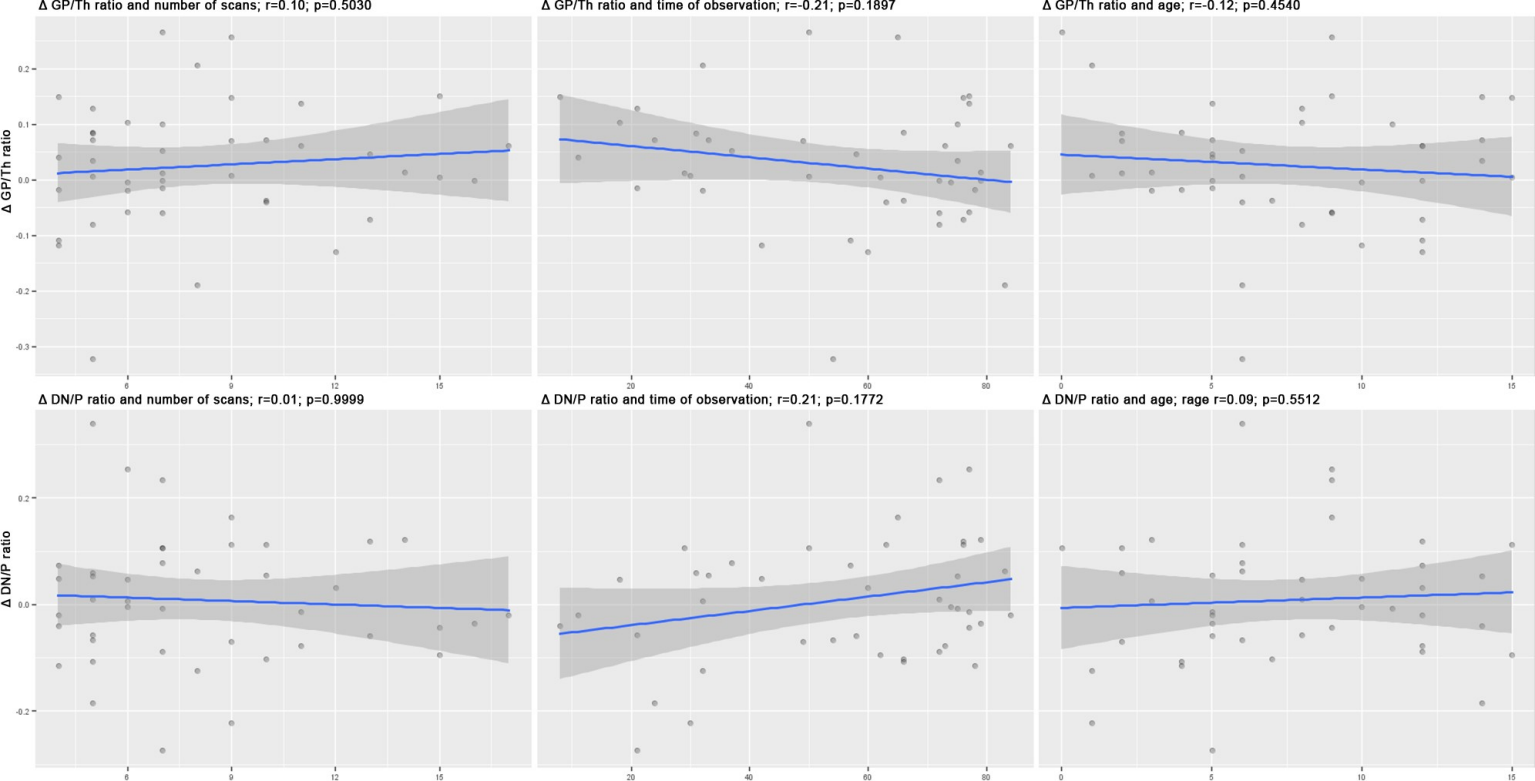
Corelations beween GP/Th ratio and the DN/P ratio vs the number of scans, time of observation and age of patients.

Correlations between Δ GP/Th ratio and age (r = -0.12; p = 0.45), time of observation (r = -0.21; p = 0.19) and the number of scans (r = 0.10; p = 0.50) were weak and insignificant. Similarly, correlations between Δ DN/P ratio and age (r = 0.09; p = 0.55), time of observation (r = 0.21; p = 0.18) and the number of scans (r = 0.01; p = 0.10) were weak and insignificant as well. Scatterplots of all correlations are presented in Fig 3.

## Discussion

The increase in signal intensity within certain brain structures after repeated injections of GBCAs has been the subject of many research projects. In our study, the comparison between pooled measurements of GP/Th ratio and DN/P ratio did not show any significant differences. Interestingly, a significant difference in GP/Th ratio was found between children older than 2 years of age and those who were 2 years of age and younger at the first scan. No other significant changes were detected.

An association between an increase in signal intensity, the number of doses, and the total cumulative dose of a contrast agent has been shown for some linear GBCAs, both nonionic and ionic and for some nonionic macrocyclic GBCAs especially in patients with multiple sclerosis. Ramalho et al. observed a significant increase in signal intensity within the dentate nucleus and the middle cerebellar peduncle as well as the globus pallidus and thalamus after 5 injections of linear nonionic gadodiamide (Omniscan) [20]. Cao et al. reported a significant increase in signal intensity in the dentate nucleus after 6 administrations of gadopentetate dimeglumine which correlated with the number of injections of this agent given to a patient [9].

Currently, a significant increase in signal intensity within brain structures related to repeated injections of macrocyclic GBCAs was reported only in 2 studies. An analysis including pediatric patients conducted by Radbruch et al. revealed no significant increase in signal intensity in 41 children with a history of at least 4 contrast enhanced MRI after the mean of 8.6 consecutive intravenous injections of gadoterate meglumine [4]. Other researchers reported similar findings in adult cohorts. The most recent study by Rossi Espagnet et al. found a significant difference between the first and last administration for both ratios: GP/Th (p = 0.004) and DN/P (p = 0.001) in the group of 50 patients after 10 administrations on average of gadoterate meglumine [8]. This study, however, was questioned by other researchers because of inconsistency and methodological limitations of the study. [21] Another study documenting enhancement of the signal intensity of the dentate nucleus on unenhanced T1-weighted MRI was performed by Bjørnerud at al. and included 17 patients with high-grade gliomas who had received 10–44 administrations of gadobutrol. They found the significant dose-dependent signal intensity increase. They also observed visually appreciable enhancement in the dentate nucleus on contrast-optimized images in 2 patients following 37 and 44 standard doses of macrocyclic GBCAs. [22] Additionally, Murata el al. found measurable amounts of various GBCAs in 5 autopsy samples from the brain tissue. The deposition of gadobutrol was higher than those of gadoteridol, gadoxetate or gadobenate [16]. These facts suggest that macrocyclic contrast agents are more stable than linear, but the risk of formation of gadolinium depositions in the brain still exists.

At birth, the human brain is unmyelinated or incompletely myelinated. Although we do not know if susceptibility to gadolinium deposition is different on the different stages of maturation of the white matter, this aspect should be addressed in the research trials. The process of myelination starts at birth. The fastest increase in the myelinated white matter volume is observed between birth and 9 months of age. Myelination should be completed at about 2 years of age. Unfortunately, there is a lack of data on the effects of exposure to GBCAs in this group of patients as most studies include older children [23]. Radbruch et al. examined a group of children aged from 3 to 17 years of age [4]. So, the study included patients with completed myelination process. The study by Flood et al. included pediatric patients older than 1 month and younger than 18 years of age, with the average age of 10.1±4.8 years in the GBCA-exposed group [3]. Hu at al. included only 1 subject who had the first examination with GBCA at the age of below 2 years [5], while Rossi Espagnet et al. excluded all children younger than 2 years [8]. Despite the number of children below the age of 2 was not large, those groups should be analyzed separately to avoid selection bias. None of the studies found any significant correlation between signal intensity and age of subjects. Our study shows that the threshold of 2 years may be important as younger children had lower baseline values of GP/Th ratio than older ones; however, further studies are needed as our study group is relatively small. We suggest that the group of patients under age of 2 years old is evaluated separately in studies assessing signal intensity measurements of brain structures.

Another important issue is the exposure to GBCAs in infants and during antennal period. Gadolinium contrast agents are water soluble molecules which can cross the placenta, thus the fetus can receive 18–30% of the administered dose which was confirmed by animal studies [24]. A dose sufficient for examination of the placenta was set at 0.1 mmol/kg [25]. The murine model showed no association between contrast enhanced MRI and both fetal development and the course of pregnancy [26]. It is worth highlighting that in the rabbit model, gadopentetate dimeglumine was detectable in fetal kidneys by imaging even 60 minutes following contrast administration [27]. The prospective study on 104 infants delivered at 24–33 weeks of gestation, who were exposed to GBCAs in utero did not present with any pathology during the neonatal period that could be associated with exposure to contrast agent and cord blood gadolinium concentration [28]. The limitation these studies is the facts that they addressed only short-term complications, and the evaluation of brain structures by MRI was not performed.

The present study has several limitations. The retrospective design did not allow for a comparison with a control group. Heterogeneity of the study group is relatively high as we included patients aged from 0 to 15 years diagnosed with various diseases, though mainly benign tumors. The control group of children not exposed to contrast agents would be of special importance to determine possible physiological changes in children during the process of myelination. Additionally, the subgroup of children aged ≤2 years was small.

Despite its limitation, this is the first study which addresses the impact of age on signal intensity in the dentate nucleus and globus pallidus. The possible differences in signal intensity during maturation of the brain suggest that assessment of signal intensity in the dentate nucleus and the globus pallidus on unenhanced T1-weighted MR images should be conducted according to age as pooled data may not produce reliable results. However, our findings should be considered preliminary, and we highly recommend further research.
